# Can Hyperpolarized Helium MRI add to radiation planning and follow‐up in lung cancer?†

**DOI:** 10.1120/jacmp.v12i2.3357

**Published:** 2011-01-31

**Authors:** Aaron M. Allen, Mitchell Albert, Hale B. Caglar, Piotr Zygmanski, Ricardo Soto, Joseph Killoran, Yangping Sun

**Affiliations:** ^1^ Department of Radiation Oncology Dana‐Farber Cancer Institute/Brigham and Women's Hospital Department of Radiology Brigham; ^2^ Women's Hospital, Harvard Medical School Boston MA USA; ^3^ Department of Radiology University of Massachusetts Medical School Worcester MA

**Keywords:** lung cancer, IMRT, functional imaging, helium MRI

## Abstract

Locally advanced non‐small‐cell lung cancer (NSCLC) is a common disease with a low overall survival even with aggressive treatments. Standard imaging (CT and PET/CT) provide no information about normal lung function. We therefore, sought to pilot HeMRI in patients with non‐small‐cell lung cancer before and after definitive radiotherapy (RT). Five patients with NSCLC receiving RT were enrolled on a prospective IRB approved study. Patients underwent CT, FDG‐PET and HeMRI before and (within 10 days) following RT. All images (CT, FDG‐PET and HeMRI) were co‐registered. The CT and PET GTVs were contoured, as well as the ventilation defects on HeMRI caused by the tumor. Patients also underwent pulmonary function tests (PFTs). Correlations between the images and PFTs were evaluated by linear regression. CT and FDG‐PET tumor volumes were highly correlated $(r^{2}=0.91$ before treatment and 0.99 following RT). There was less correlation between HeMRI and CT or PET ($r^{2}=0.67$ (CT) and 0.38 (PET)) prior to treatment and 0.27 following RT). However, HeMRI volumes correlated very well with FEV1, both prior to and following RT. $(r^{2}=0.89$ and 0.83, respectively). ${}^{3}\text{Helium}$ MRI scanning is feasible in NSCLC before and after treatment. HeMRI provides important functional information in addition to CT and CT/PET scanning.

PACS number: 87.55.D‐

## I. INTRODUCTION

Locally advanced non‐small‐cell lung cancer (NSCLC) represents a therapeutic challenge for oncologists today. Chemoradiation or chemoradiation followed by surgery have produced a 30% 5‐year overall survival.^(^ ^1^ ^,^ ^2^ ^)^ One of the strategies that has been attempted with early promising results is dose escalated radiotherapy.^(^ ^3^ ^,^ ^4^ ^)^ However, two cooperative group dose escalation trials have found that 74 Gy is the maximum achievable dose with concurrent chemotherapy because of normal tissue toxicity constraints.^(^ ^5^ ^,^ ^6^ ^)^ If further gains in local control, through dose escalation, are to be made new avenues of reducing dose to the normal tissues must be found.

One option to improve the therapeutic window, is to preferentially spare sections of the lungs that are most critical to the patient's overall lung function, and preferentially treat areas of the lung that are nonfunctioning.

How can we determine which sections of the lungs are most critical to the patient's overall functional pulmonary capacity? Investigators have evaluated SPECT (single positron emission computed tomography) scans using lung perfusion as functional surrogate of lung function, and then used modern planning techniques to plan radiotherapy based on these scans.^(^ ^7^ ^,^ ^8^ ^)^ Another option is a new technique called Hyperpolarized ${}^{3}\text{Helium}$ MRI. (HeMRI) Proton (^1^H) MRI has not been used extensively in lung imaging due to the poor resolution of alveolar structure on MRI. However, when helium gas is inhaled it remains within the lung tissue without diffusing into the blood. When helium undergoes polarization, it aligns the spins of all the helium molecules, and then one can produce a vivid detailed image of the functional ventilation space of the lungs with MRI scanning. ${}^{3}\text{HeMRI}$ has shown to be highly accurate in describing and quantifying lung function in many pulmonary diseases.^(^ ^9^ ^–^ ^12^ ^)^ In addition, HeMRI has been used in radiation therapy planning. Ireland et al.^(13)^ showed in a study of six patients that fusion of HeMRI images with treatment planning CT was feasible, and that IMRT plans using the HeMRI images to designate functional and nonfunctional lungs could be created to limit dose to “functional” lung. In preclinical models, Ward et al.^(14)^ have shown that after high‐dose radiation to the rat lung (5×8 Gy), changes seen on HeMRI correlate well with radiation induced lung fibrosis. Recently, Cai et al.^(15)^ have shown that after delivering stereotactic‐like RT (20 Gy×3) to rat lungs, correlated changes were seen on perfusion MRI but not on HeMRI.

Since ${}^{3}\text{HeMRI}$ produces both 3D anatomic ventilation map as well as known correlation with functional pulmonary function, we sought to examine its use as an addition to traditional imaging in patients with lung obstruction/destruction due to lung cancer. We report our preliminary experience with ${}^{3}\text{HeMRI}$ both before and after RT for NSCLC.

## II. MATERIALS AND METHODS

### A. Patient selection

Newly diagnosed NSCLC patients treated with radiation therapy (RT) at the Dana‐Farber/Brigham and Women's Hospital Cancer Center (DF/BWHCC) were eligible to enroll on this prospective, IRB‐approved, pilot imaging trial. Patients with previous surgery or prior anticancer therapy were not eligible. Patients had to have good performance status (0 or 1), without significant weight loss (< 10% body weight) to enroll.

### B. Pretreatment evaluation

All patients underwent routine physical examinations, blood work and staging studies consistent with routine practice at DF/BWHCC. In addition, each patient underwent full pulmonary function testing (PFT) including forced expiratory volume (FEV1), functional vital capacity (FVC) and carbon dioxide diffusion testing (DLCO).

### C. Radiation planning and treatment

Each patient underwent CT simulation (free breathing) with Vac‐loc and T‐bar immobilization (Med‐Tec Civco, Orange City, IA). Axial CT and PET/CT images were transferred to ECLIPSE (Varian, Palo Alto, CA) treatment planning system and three‐dimensional conformal radiotherapy (3DCRT) was planned without using information provided by the HeMRI images. Total dose was at the discretion of the treating physician but was typically between 66–70 Gy. Treatment was delivered on Varian Clinac 2100Ex linear accelerator, once daily, for the duration of the treatment.

### D. FDG‐PET scanning

Following simulation, all patients were scheduled to undergo whole‐body ${}^{18}\text{FDG}‐\text{PET}/\text{CT}$ (PET) scanning prior to, and after completion of (within ten days), RT. PET/CT scans were done using an integrated PET/CT scanner (Discovery LS GE Medical Systems, Milwaukee, WI). Patients were scanned prior to treatment on a flat tabletop with the simulation immobilization to ensure appropriate positioning for image fusion. Image fusion was performed using the Advantage SIM MD workstation (GE Medical Systems, Milwaukee, WI). Two separate physicians (both with over five years of thoracic RT experience) evaluated the PET/CT fusion. No large deviations (> 10 mm) were seen with the image fusion. Tumor volumes were contoured on both CT and PET images. Tumor volumes were recorded in cm^3^. The post‐treatment PET and CT images were also fused and tumor volumes were recontoured. A single physician performed all the contouring and then a second physician reviewed the contours for consistency. FDG‐PET‐positive volumes were clinically defined with a lower threshold of SUV > 3 (per RTOG standards).

### E. Hyperpolarized Helium MRI

MRI was carried out on a General Electric Signa LX 1.5‐T MRI scanner (GE Medical, Milwaukee, WI). For HP ${}^{3}\text{He}$ MRI, a heterodyne system was appended to the MRI system to enable imaging at the ${}^{3}\text{He}$ Larmor frequency. Signal was collected using a flexible quadrature wrap‐around lung coil (Clinical MR Solutions, Brookfield, WI) tuned to the ${}^{3}\text{He}$ frequency. ${}^{3}\text{He}$ was hyperpolarized via collision spin exchange with optically pumped rubidium using a ${}^{3}\text{He}$ polarizer built in‐house, achieving polarizations between 10% and 20%. For each scan, 1 L of approximately $33\%\;{\text{HP}}^{3}\text{He}–67\%N_{2}$ mixture was administered for the subject to inhale. Subjects were positioned supine with arms extended to mimic simulation procedures. They were then instructed to inhale the 1 L of gas and hold their breath. Each scan began immediately upon breath‐hold. The scans used a fast gradient echo pulse sequence acquiring coronal projection images with the following parameters: 46 cm FOV, 0.75 Phase FOV, 128×256 matrix, 13 mm slice thickness, 0 mm gap between slices, 31.25 kHz bandwidth, 14°–18° flip angle, TE/TR 1.228 ms/50–75 ms, and interleaved data acquisition. Scans ranged from 5–10 seconds, depending on the anterior–posterior depth of the lungs, which may require 9 to 14 slices to encompass. The acquired data were then zero‐padded to produce images with 256×256 pixels.

### F. Helium MRI contouring

Axial helium MRI images were fused with CT datasets using the GE Advantage SIM MD software (GE Medical). A point‐to‐point fusion was done relying on the carina and main bronchi, and was manually fused the HeMRI scans with CT simulation scans. Each image fusion was reviewed by a second physician for consistency. The external boundaries of the lung were defined on the fused axial CT scans and total lung volumes were then determined on the Helium MRI scans. The HeMRI tumor volume (total lung volume‐Helium‐3 MRI lung volume) was contoured on the axial slices and a 3D structure was created. Small ventilation defects $\left(<10\;{\text{cm}}^{3}\right)$ distant to the site of the primary tumor volume were not considered in this analysis.

### G. Imaging timeline

Patients were scheduled to undergo CT, full‐body FDG‐PET and Hyperpolarized Helium MRI at two time points: prior to radiotherapy, and immediately following the completion of therapy (within 10 days).

### H. Pulmonary function tests

At each of the imaging time points as outlined above, each patient underwent full pulmonary function tests (PFTs). All pulmonary function tests were performed at a single laboratory at the Brigham and Women's Hospital. The tests included forced expiratory volume in one second (FEV1), functional vital capacity (FVC), carbon dioxide diffusion capacity (DLCO). The DLCO values were corrected for the patient's hemoglobin content. The PFTs were recorded as absolute values, as well as in percent predicted.

### I. Analysis

Volume measurements of CT, PET and Helium MRI volumes were done using the Advantage SIM MD software. These values were exported to Microsoft Excel where linear regression analysis was performed to compare the volumes (CT, PET, HEMRI) to each other and to the PFT values.

## III. RESULTS

### A. Clinical parameters

Five patients with NSCLC were enrolled on this pilot study. An additional patient was screened but did not enroll on the protocol secondary to progression of disease. There were four males and one female. Median age was 61 years old (range from 54–70). Four patients had stage IIIA NSCLC and one patient had stage I NSCLC. The median dose of radiotherapy received was 68 Gy (range from 54–70). Four patients received concurrent chemotherapy together with radiation therapy. All patients completed their prescribed dose of therapy. All imaging tests and PFTs were completed in all five patients at pretreatment and immediately following completion of therapy.

**Figure 1:**
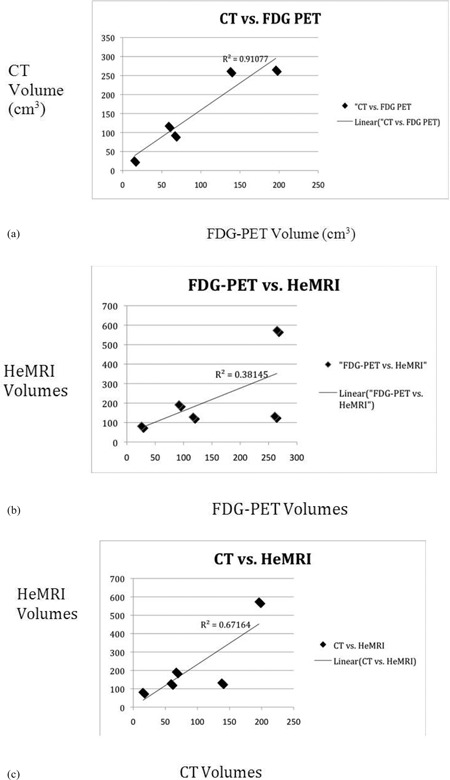
Plots comparing tumor volumes prior to RT of: a) FDG‐PET vs. CT, b) FDG‐PET vs. HeMRI, c) CT vs. HeMRI. Correlation coefficients and trend line given by linear regression.

The median baseline CT‐based tumor volumes or GTV (gross tumor volume) was 117 cm^3^ (range from 26 to 265 cm^3^). FDG‐PET volumes were smaller, with median volume of 67 cm^3^ (range 15 – 196 cm 3). The initial median Helium MRI volume was 132 cm^3^ (range $81–573\;{\text{cm}}^{3}$). The complete initial volumes can be seen in Table 1. When comparing CT, PET and Helium MRI initial volumes there was an excellent correlation between CT and PET volumes $(r^{2}=0.91$, see Fig. 1(a). However, the correlation between CT and Helium MRI or PET and Helium MRI was not nearly as strong $(r^{2}=0.67$ and 0.38, respectively, Fig. 1 (b) and (c)) as expected.

**Figure 2:**
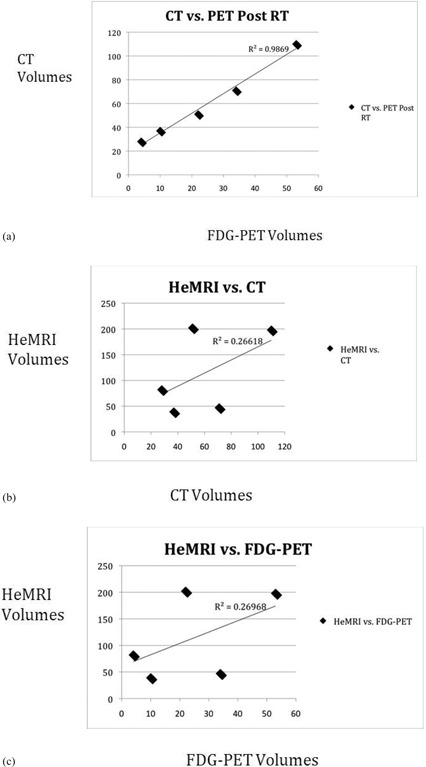
Plots comparing tumor volumes following RT of: a) FDG‐PET vs. CT, b) CT vs. HeMRI, c) FDG‐PET vs. HeMRI. Correlation coefficients and trend line given by linear regression.

**Figure 3(a):**
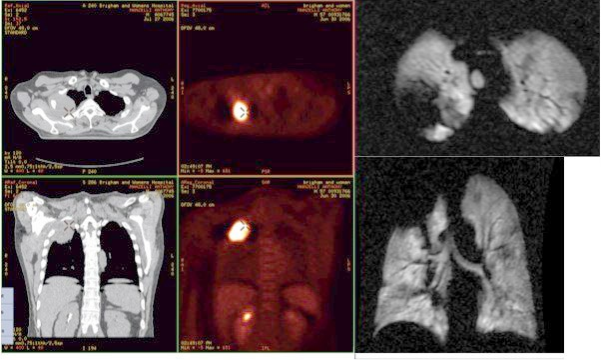
Patient #1 – right upper lobe mass demonstrated on fused PET/CT, with axial (upper) and coronal (lower) slices of Helium MRI.

**Table 1:** Tumor volumes in (cc) prior to and after RT.

*Patient* #	*CT Volume (pre‐XRT)*	*CT Volume (post‐XRT)*	*PET Volume (pre‐XRT)*	*PET Volume (post‐XRT)*	*HeMRI Volume (pre‐XRT)*	*HeMRI Volume (post‐XRT)*
1	261	71	138	34	132	47
2	265	110	196	53	573	198
3	92	51	67	22	197	202
4	117	37	59	10	128	39
5	26	28	15	4	81	82

### B. Response to therapy

In 4/5 patients, tumor shrinkage was seen in response to radiotherapy, based on CT tumor volumes. The median decrease was 59% (range 73% decrease to 8% increase). The median percent volume change based on FDG‐PET was a 73% decrease (range from 67%–83%). The changes in the Helium MRI volumes were more varied. The median change in Helium MRI volumes was a 64% decrease (range 70% decrease to 5% increase).

Comparisons of the CT and PET responses again showed excellent correlation. Figure 2 shows the relationship between the post‐therapy CT and PET volumes with a correlation of $r^{2}=0.99$ (Fig. 2(a). Conversely, the relationship between HeMRI and CT and PET decreased with an $r^{2}=0.27$ for both comparisons (Fig. 2(b).

A more thorough examination of the disagreement between helium MRI and standard imaging (PET and CT) reveals some interesting points. In one case (Fig. 3(c), the entire left upper lobe had been obstructed by the tumor such that, after response to radiation, the HeMRI volume decreased substantially secondary to both tumor shrinkage and volume re‐expansion. In another case (Fig. 3(e). the tumor volume decreased on CT and PET, indicating a response. Yet, the helium volume was unchanged suggesting either a smaller response than seen on CT or decreased ventilation secondary to radiotherapy. This patient following completion of protocol treatment was taken to surgery and underwent pneumonectomy. The histological description of the tumor size was nearly identical to the measured size on CT but had an attached fibrous bridge extending towards the pleural margin corresponding to the defect seen on the helium scan after treatment (Fig. 3(f).

**Figure 3(b):**
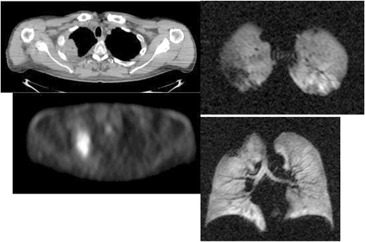
Following XRT, response is seen on CT, PET and Helium MRI.

**Figure 3(c):**
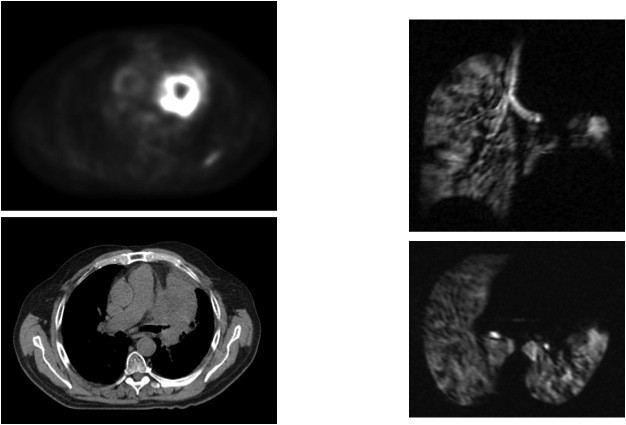
Patient #2 – obstructive left upper lobe mass with complete loss of ventilation on Helium MRI.

**Figure 3(d):**
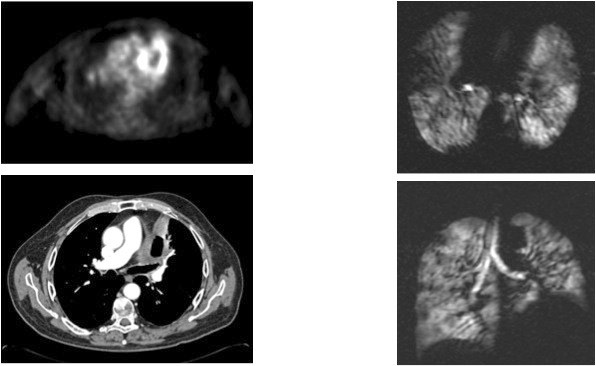
Response after XRT – cavitation is seen on CT and response on PET. Interestingly obstruction nearly completely resolved on Helium MRI.

**Figure 3(e):**
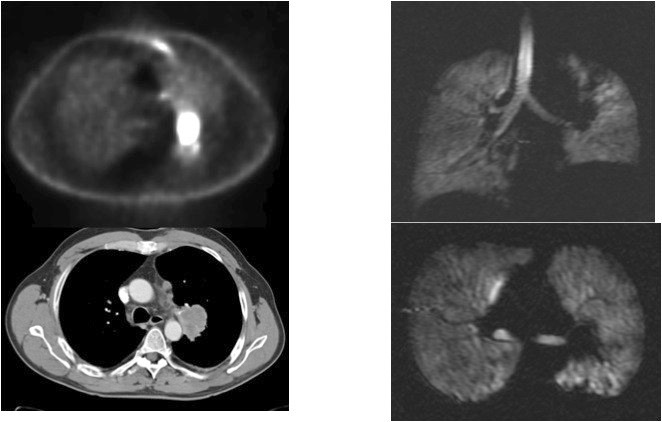
Patient #3 – with central partially obstructing mass on CT, PET and Helium MRI.

**Figure 3(f):**
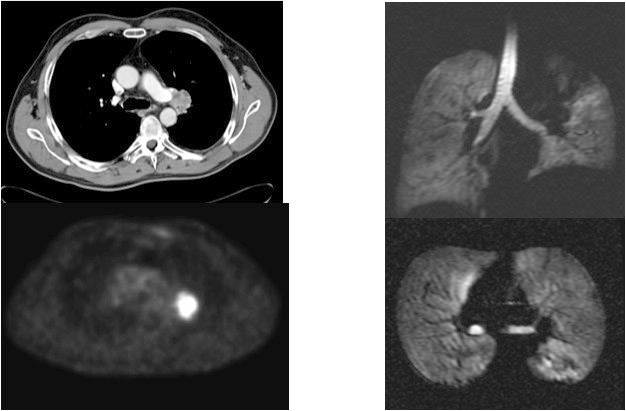
After XRT, mass undergoes PR and lung partially re‐inflates.

The remaining cases are illustrated in (Figs. 3g), (h), and (i).

**Figure 3(g):**
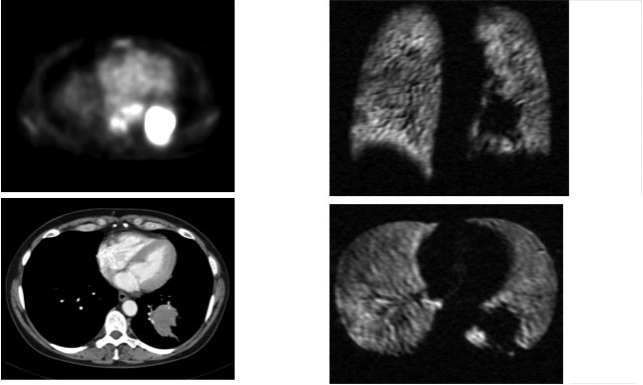
Following XRT, excellent response is seen on all three modalities.

**Figure 3(h):**
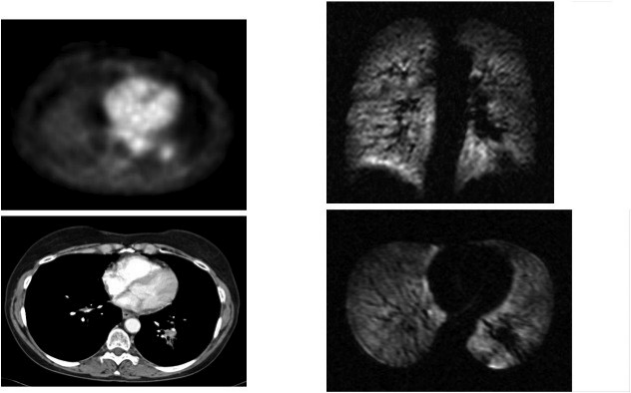
Patient #5 – Small left lingular mass seen on CT, PET, HeMRI.

**Figure 3(i):**
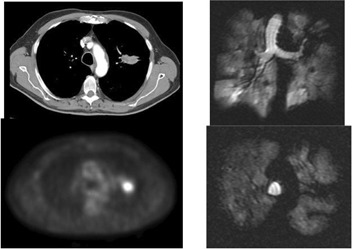
Minor response to XRT – stable images on all three modalities.

**Figure 3(j):**
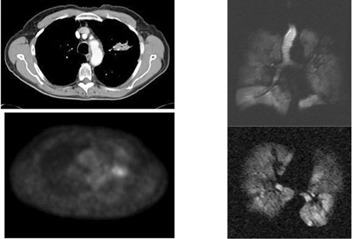
Patient #4 – left lower lobe space occupying lesion seen on CT, PET and MRI.

### C. Pulmonary function results

Baseline PFTs (prior to RT) were within normal limits in most of the patients with median FEV1 and FEV1 predicted of 2.5 liters and 96%, respectively. The baseline DLCO values were slightly lower with a median DLCO of 18.8 and percent predicted DLCO of 77%. When one views the results for all patients in Table 2, it can be seen that patients #2 and #3 who had bronchial obstruction secondary to tumor, had impaired lung function tests.

**Table 2:** Pulmonary function tests before and after radiotherapy.

*Patient* #	*Pre‐XRT FEV1*	*Post‐XRT FEV1*	*Pre‐XRT %FEV1*	*Post‐XRT %FEV1*	*Pre‐XRT DLCO*	*Post‐XRT DLCO*
1	3.37	3.52	96	100	21	20.94
2	1.51	1.66	51	55	13.7	12
3	2.6	2.6	76	76	20.9	19.8
4	2.2	2.4	100	111	18.8	15.7
5	2.5	2.4	105	102	18.2	18.8

When the Helium MRI volumes are compared with the patient's pulmonary function tests, a close correlation was seen. The initial percent predicted FEV1 was highly correlated with the initial volume $(r^{2}=0.89$, Fig. 4(a) and following RT, the Helium MRI and PFTs maintained a good correlation with $r^{2}=0.83$ (Fig. 4(b).

**Figure 4(a):**
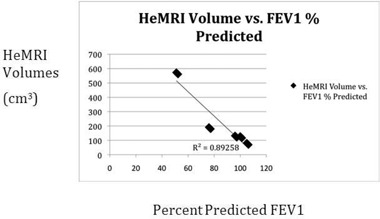
Plot of HeMRI volume pre‐RT vs. percent FEV1 predicted.

**Figure 4(b):**
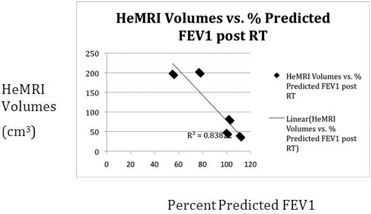
Plot of HeMRI volume post‐RT vs. percent FEV1 predicted.

## IV. DISCUSSION

Imaging has an important role to play in the radiotherapeutic management of NSCLC. CT has been – and continues to be – the backbone of thoracic imaging. In addition, the use of FDG‐PET has become common practice due to its ability to add valuable information for both staging and tumor definition purposes. However, just as PET helps the radiation oncologist locate the tumor precisely, Hyperpolarized ${}^{3}\text{Helium}$ MRI has the ability to help radiation oncologist understand the status of the normal lung tissue as they approach the radiation planning and follow‐up of these patients.

Our pilot study demonstrated that HeMRI can produce clear 3D axial and coronal images of the lungs of patients with NSCLC. Unlike CT or PET, HeMRI was able to define areas of lung obstruction before treatment and restoration of lung expansion following treatment (Fig. 3(c), (d)). These scans, unlike CT and PET, also correlated well with a known parameter of lung function FEV1.

What is the significance of these findings? Radiation planning for lung cancer requires radiation oncologists to make a careful evaluation of the normal tissue constraints on the lungs to avoid radiation pneumonitis. Dose volume limits commonly used such as V20 (the volume of lung receiving 20 Gy or more) are based on the volume of lung tissue that has the Hounsfield units of aerated lung on CT (that appear to be functional lung tissue on CT). The implications of this information are that obstructed or collapsed lung is not typically considered as part of the total lung volume for the purposes of radiation planning.

Based on the results of our study, we have shown two important limitations of this method. First, we showed in patient #3 (Fig. 3(c) that the entire upper lung distal to the obstruction on HeMRI is nonfunctional. However, on the CT simulation of this patient's lung prior to radiation, although there is a focal area of atelectasis, the lung superior (cranial) to the atelectasis lung appears aerated (i.e., has the CT imaging characteristics of functional lung) and was included in the lung volume for purposes of the calculation of V20. If HeMRI was used for RT planning, this area of lung which, based on Fig. 3(c) is nonfunctional, would have been excluded from calculations of total lung volume and consequently, perhaps, a more conservative radiation plan would have been created. On the other hand, following RT when the tumor shrinks and the lung re‐expands, the amount of functional lung seen on HeMRI significantly increases ((Figs. 3d), 3(f)). The result is that the initial CT simulation is fraught with significant limitations in cases of lung obstruction due to tumor. Consequently, the estimation of the normal lung volume is limited. In a larger cohort, a 3D functional ventilation map of the lungs would certainly be helpful to radiation oncologists and a further study of HeMRI scanning could accomplish this.

Helium MRI is also not the first functional imaging technique to try to examine the normal lung tissue in the setting of radiotherapy. Single positron emission tomography (SPECT) studies have been done in thoracic radiotherapy for a number of years. Marks et al.^(16)^ demonstrated a 40 Gy dose as the threshold that corresponds to changes in perfusion on SPECT scans. These data led to creation of dose functional histograms that identified novel DVH parameters, which included functional imaging data into dose constraints.^(17)^

More recently, the Duke group and others have used SPECT scans to serve as a guide for IMRT. Studies by Shioyama et al.,^(18)^ McGuire et al.,^(19)^ and Laverenkov et al.^(8)^ all showed the ability to avoid “nonfunctional” lung tissue with IMRT and thereby improve DVH metrics. However, SPECT scans as done in these studies all examine pulmonary perfusion as a measure of lung function. Although, there is good evidence from the surgical literature that perfusion SPECT scans correlate well with regional lung function, they do not provide the level of anatomic resolution needed to measure tumor volume defects nor can they quantitate the volume of the defects down to the cm^3^ level.^(20)^ In a recent literature review, the spatial resolution of SPECT was assessed to be half as good as CT or even PET imaging. This is because of source discrimination, as well as respiratory motion effects and volume averaging issues, which do not exist to the same extent on CT or FDG‐PET images.^(21)^

Our study is one of the first to examine post‐RT changes in humans with HeMRI. The results of our study, although limited by being based on small numbers, raise some interesting questions:

1. Is the delineation of the normal lung volume for DVH and pneumonitis prediction on CT reliable when a large portion of the lung may is functionally obstructed, as is seen in patient #2 in our study?
2. Or conversely, is it appropriate to use functional imaging SPECT or perfusion MRI or HeMRI combined with IMRT to preferentially deliver dose to “nonfunctioning” areas of the lung when these areas may return to function following tumor shrinkage or removal of bronchial obstruction following treatment?

Our study cannot answer these questions definitively but raises issues for further study. We believe that a reliance on CT alone for characterization of the normal lung tissue is not sufficient. However, perfusion scans alone as an addition to CT may not include the whole functional picture, and a combination of HeMRI and SPECT or perfusion MRI may be a good direction for further study.

In future studies, we would recommend the following directions for the use of HeMRI in the planning and follow‐up of patients with lung cancer receiving radiation therapy. Specifically, we would recommend the use of two volumes for calculating V20 and MLD to be used in planning and optimization. The first lung volume would include the classic total lung volume‐GTV as outlined in the literature. The second volume, based on HeMRI, would be total functional lung volume excluding both tumor and nonventilated lung tissue as seen on HeMRI.

In follow‐up, HeMRI may also have an important role to play. Since HeMRI correlates extremely well with FEV1 (both in our study and in other studies of HeMRI) HeMRI could be used monitor patients for pulmonary deterioration following treatment. In this case, the added benefit of HeMRI over PFTs and conventional imaging would be to indentify the anatomic location causing the decrease in pulmonary function. For example, in Fig. 3(c) we see that the decrease in PFTs is due to obstruction plus tumor mass, as compared to Fig. 3(j) where the tumor obstruction is the only cause of functional lung tissue.

Our study has some limitations. Because our study was a pilot study intended to explore the value of HeMRI in NSCLC, only five patients were scheduled to be enrolled. In addition, we did not use a measure of perfusion scanning to accompany HeMRI scanning. Finally, we performed our post‐RT scans and PFT at the conclusion of therapy. It is likely that, if these tests had been done 6 or 12 months post‐RT, additional important data may have been obtained.

## V. CONCLUSIONS

In summary, Helium MRI scanning provides a new outlook of the normal lung tissue of patients with NSCLC undergoing RT. The HeMRI volumes were closely correlated with FEV1 and provided additional information over CT and FDG‐PET scans. Further study will be needed with this novel technology to determine its impact for radiologists and radiation oncologists.
